# Recent fire in a Mediterranean ecosystem strengthens hoverfly populations and their interaction networks with plants

**DOI:** 10.1002/ece3.9803

**Published:** 2023-02-07

**Authors:** Georgios Nakas, Aphrodite Kantsa, Ante Vujić, Mark C. Mescher, Consuelo Μ. De Moraes, Theodora Petanidou

**Affiliations:** ^1^ Department of Geography University of the Aegean Mytilene Greece; ^2^ Department of Environmental System Sciences ETH Zürich Zürich Switzerland; ^3^ Department of Biology and Ecology, Faculty of Sciences University of Novi Sad Novi Sad Serbia

**Keywords:** disturbance, mediterranean ecosystems, pollination, post‐fire recovery, syrphidae, wildfires

## Abstract

Fire affects many critical ecological processes, including pollination, and effects of climate change on fire regimes may have profound consequences that are difficult to predict. Considerable work has examined effects of fire on pollinator diversity, but relatively few studies have examined these effects on interaction networks including those of pollinators other than bees. We examined the effects of a severe wildfire on hoverfly pollinators in a Mediterranean island system. Using data collected over 3 consecutive years at burnt and unburnt sites, we documented differences in species diversity, abundance, and functional traits, as well as hoverfly interactions with flowering plants. Hoverfly abundance and species richness peaked during the first post‐fire flowering season (year 1), which coincided with the presence of many opportunistic species. Also in year 1, hoverfly pollination networks were larger, less specialized, more nested, and less modular at burnt (vs. unburnt) sites; furthermore, these networks exhibited higher phylogenetic host‐plant diversity. These effects declined over the next 2 years, with burnt and unburnt sites converging in similarity to hoverfly communities and interaction networks. While data obtained over 3 years provide a clear timeline of initial post‐fire recovery, we emphasize the importance of longer‐term monitoring for understanding the responses of natural communities to wildfires, which are projected to become more frequent and more destructive in the future.

## Graphical abstract

We examined the effects of a severe wildfire on hoverfly pollinators in a Mediterranean island ecosystem in Greece. Using data collected over three consecutive years at burnt and unburnt sites, we documented differences in species diversity, abundance, and functional traits, as well as hoverfly interactions with flowering plants. Our work provides a clear timeline of initial post‐fire recovery, and emphasizes the importance of longer‐term monitoring for understanding the responses of natural communities to wildfires, which are projected to become more frequent and more destructive in the future.

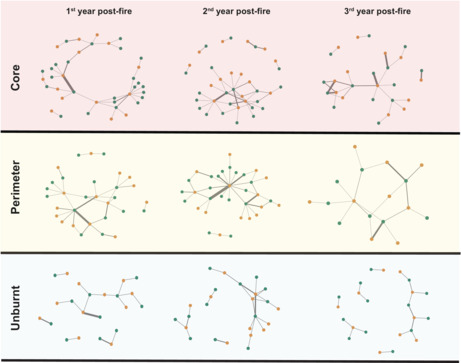

## 1 INTRODUCTION

Fire influences many important ecosystem patterns and processes, including vegetation structure, the carbon cycle, and climate at regional and global scales (Bowman et al., 2009). Especially in the Mediterranean, fire has been considered a key factor that historically shaped ecosystems and drove several plant adaptations (Keeley et al., 2011; Pausas & Keeley, 2009). On the other hand, under the influence of climate change, fires are expected to become more severe, more frequent, with longer seasons, and more challenging to control (Flannigan et al., 2013; Jolly et al., 2015). All these bear potentially profound consequences for ecosystems that are difficult to anticipate. Improved knowledge regarding the effects of different fire regimes on biological communities is thus critical for efforts to predict and manage the ecological consequences of wildfires in future climate conditions (Brown et al., 2017).

Over the last few decades, substantial progress has been made to track the response of pollinator diversity to wildfires (e.g., Adedoja et al., 2019; Burkle et al., 2019; Carbone et al., 2019, 2017; Lazarina et al., 2019; Mason et al., 2021; Moretti et al., 2009; Peralta et al., 2017, 2001; Potts et al., 2003; Viljur et al., 2022). This work has shown that fire can affect insect pollinators in numerous ways other than via direct mortality (Brown et al., 2017). For example, such indirect impacts can be mediated via changes in soil conditions (Carbone & Aguilar, 2017; Certini, 2005), plant community composition (Lavorel & Garnier, 2002), food availability (Potts et al., 2004; Simanonok & Burkle, 2020), and nesting resources (Moretti et al., 2009; Simanonok & Burkle, 2019). However, there are still crucial gaps in our knowledge regarding fire effects on pollinator communities and plant–pollinator interactions. For example, work to date has focused heavily on bees, largely ignoring other pollinator guilds. Furthermore, while many studies have recorded effects on species diversity, relatively few of them have examined pollinator's functional traits; moreover, there is a pronounced lack of studies examining the effects of fire on plant–pollinator interaction networks, especially in the Mediterranean area.

With respect to taxonomic biases, the majority of studies examining fire effects on pollinators address Hymenoptera (see Carbone et al., 2019 and literature included therein), while only a few studies have focused on Diptera, including hoverflies (but see Johansson et al., 2020; Lazarina et al., 2019). Yet, hoverflies (Diptera: Syrphidae) are among the most important insect pollinators in many habitats (Doyle et al., 2020; Vujić et al., 2020). In Europe, more than 900 hoverfly species are known and more than half of which occur in the Mediterranean area (with 418 species reported in Greece alone), where the rate of discovery of new species is the highest in Europe (Vujić et al., 2020). Despite their importance, our knowledge of the response of hoverflies to disturbance is geographically fragmented and far from complete. Long‐term monitoring of insect communities has shown declines in hoverfly abundance and/or species richness, for example, in NW Germany (Hallmann et al., 2021), the UK (Biesmeijer et al., 2006), or the Netherlands (Barendregt et al., 2022). On the other hand, hoverflies can be less susceptible to habitat fragmentation compared with other pollinators, such as bees (Jauker et al., 2009), and may even benefit from common disturbances, including moderate levels of grazing in the Mediterranean region (Lázaro, Tscheulin, Devalez, Nakas, & Petanidou, 2016). This implies some degree of hoverfly resilience to disturbance and suggests that these insects might act as surrogate pollinators in cases where bees are absent or in decline (Biesmeijer et al., 2006; Jauker & Wolters, 2008; Pérez‐Bañón et al., 2003, 2007; Rader et al., 2013).

Although recent work has highlighted the importance of functional trait diversity within biological communities in the context of disturbance (de Bello et al., 2010; Violle et al., 2017), empirical work on the effects of fire on the functional traits within pollinator communities remains notably scarce. Pausas and Parr (2018) emphasized that, contrary to many plant species, most animal trait adaptations to fire are rather behavioral and not morphological (for a review on insect adaptations, see Swengel, 2001). For example, nesting strategies can predict species' post‐fire survival: the larvae of the majority of ground‐nesting bees (especially those building deep nests) are more resilient to fire than bees with other nesting preferences, simply because they can survive soil heating (Cane & Neff, 2011). In a comparative study of burnt habitats in Switzerland and Israel, Moretti et al. (2009) showed that fire consistently selected short‐tongued bees with low mobility and late phenology in both countries; however, fire predicted the nesting resources of the pioneer species only in Switzerland.

It is important to highlight that, in contrast to bees, hoverflies exhibit remarkable diversity in functional traits both at the adult and at the larval stages (Miličić et al., 2021; Vujić et al., 2020), which may well have important implications for responses to fire or other environmental stressors. For instance, hoverfly larvae can be active in various habitats and microsites (e.g., plant stems, bulbs, water bodies, etc.) and show diverse trophic habits [carnivores, herbivores, or microphages (Speight et al., 2020; Vujić et al., 2020)] that are sometimes completely different than those of the adult stages, complicating efforts to predict which traits of a life stage may be favored or disfavored by fire.

Finally, but critically, our current lack of knowledge regarding effects of fire and other disturbances on plant–pollinator interactions and the structure of pollination networks is a major barrier to predicting the consequences of changing fire regimes at the community level. The few available studies focus almost exclusively on bees and they present contrasting results. Ne'eman et al. (2000) found that fires affect bee visitation rates on four important Mediterranean plant species, resulting in lower fruit set in the burnt sites. Other studies found no significant difference in terms of visitation rates and fruit set when burnt and unburnt areas were compared (García et al., 2018; Potts et al., 2001). Regarding pollination networks, a few studies (e.g., Peralta et al., 2017; Adedoja et al., 2019; Baronio et al., 2021) have shown that fires may affect network structure, again with inconsistent results.

To address the limitations of our current knowledge, this study examines the responses of species diversity, abundance, and functional traits of hoverflies, as well as their interactions with flowering plants using data collected systematically for 3 consecutive years after a severe wildfire event in a Mediterranean island ecosystem. It should be noted here that all studies we know of have substituted “space‐for‐time” (e.g., Lazarina et al., 2017; Moretti et al., 2009; Peralta et al., 2017; Potts et al., 2003). Being migratory, many hoverfly species are able to fly long distances in order to exploit new resources; in addition, many are considered generalist (polylectic) flower visitors (Jia et al., 2022; Lucas et al., 2018). Here, we predict that, if compared with unburnt sites, communities recently exposed to fire exhibit (1) relatively higher abundance and diversity of hoverflies, (2) higher abundance of hoverfly species with specific functional traits (e.g., migratory), (3) more diverse communities of hoverfly host plants, and (4) larger and less specialized networks. Furthermore, we foresee that such fire effects decline over time, with burnt and unburnt communities becoming more similar in the following years.

## 2 METHODS

### 2.1 Study sites

**FIGURE 1:**
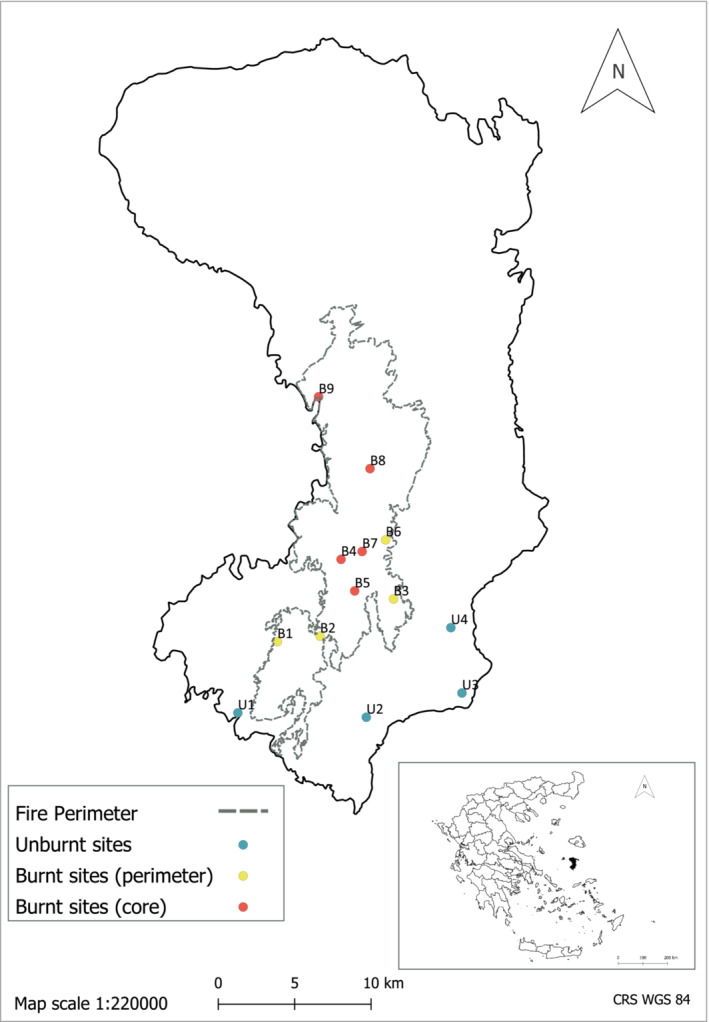
The study sites on Chios Island (for details, see Table S1). Data for the perimeter of the fire have been provided by the Forest Service of Chios Island.

The study was conducted on Chios Island, North Aegean, Greece (Figure 1), following the fire event of 18–28 August, 2012, that burnt ca. 14,800 hectares in the western, central, and southern part of the island (estimation by the Forest Service of Chios; Figure 1). In November 2012, we selected 13 study sites (hereafter referred to as “sites”) (Figure 1 and Table S1) after thorough in situ survey including both the burnt and the surrounding unburnt areas. The selected sites included: four unburnt phrygana sites dominated by entomophilous low shrubs (viz. *Salvia fruticosa*, *Cistus* spp., *Satureja thymbra, Genista acanthoclada,* and *Thymbra capitata*); and nine burnt sites with burnt vegetation including sporadic intact unburnt patches. Pre‐fire habitat types of the burnt sites were phrygana, pine forests (dominated by *Pinus brutia*), maquis (dominated by *Arbutus unedo, A. andrachnae*, and *Quercus* spp.), and shrub cultivations (viz. olive or mastic groves with olive trees, *Olea europaea,* and mastic tree, *Pistacia lentiscus* var. *chia*, respectively). Each site covered at least 0.4 ha having a minimum distance of 1.5 km from the nearest site (Figure 1 and Table S1). Burnt sites were further categorized in two groups: perimeter sites (four sites, with a < 500 m distance from the fire border/perimeter) and core sites (five sites, with a > 900 m distance from the fire border/perimeter) (Figure 1 and Table S1). The distinction between core‐ and perimeter‐burnt sites stemmed from previous studies indicating that hoverflies respond to fires at a range 400–1000 m (Lazarina et al., 2019) or can carry pollen for up to 400 m (Rader et al. (2011).

### 2.2 Insect diversity and visitation sampling

Sampling was conducted during the first 3 post‐fire years, 2013, 2014, and 2015 (hereafter year 1, year 2, and year 3, respectively). In each site and year, we performed three monthly sampling sessions (rounds) a month apart, from late March to late June, covering the main flowering season. Insect collection was always conducted by the same trained collector using both pan‐traps and hand netting, a combined methodology proved to be the most suitable for assessing pollinator richness in the Mediterranean (Nielsen et al., 2011; Lázaro, Tscheulin, Devalez, Nakas, & Petanidou, 2016; Minachilis et al., 2020; Lázaro et al., 2021). For the pan‐trap collections, 10 triplets of UV‐bright pan‐traps (500 mL plastic bowls) of yellow, blue, and white color were used (Nielsen et al., 2011). The triplets were placed with a 10 m minimum distance between each other, each bowl filled with ~350 mL of water with a drop of dishwashing detergent added to reduce surface tension, and left in situ for 48 h until collection. Hand‐netting survey consisted of 2 h of random walking per site and round and aimed at collecting (unidentifiable on the wing) or recording (readily identifiable) all insects visiting the flowers and touching their reproductive organs. Samplings were carried out during the peak of pollinator activity, i.e., between morning and early afternoon hours (9:00 am to 4:30 pm) under good weather conditions (sunny, temperature between 15 and 38°C, and wind speed <3.5 m/s).

Insects were identified at the Laboratory of Biogeography & Ecology, University of the Aegean, and the Department of Biology and Ecology, University of Novi Sad. All collected specimens were identified to species level, apart from 11 specimens that were identified as three morphospecies (hereafter referred to as distinct species). The insects are deposited in the *Melissotheque of the Aegean* of the University of the Aegean (Petanidou et al., 2013) and the Department of Biology and Ecology of the University of Novi Sad.

### 2.3 Flower cover

Floral resources at each site and round were monitored by measuring flower cover. Within each site, 25 squares of 1 m^2^ were randomly selected, and the total number of functional reproductive units per plant species (i.e., flowers or inflorescences depending on the species, hereafter referred to as “flowers”) was counted in each square. All plant specimens were identified to species level except for two that were described as morphospecies (*Orobanche* spp. and *Taraxacum* spp., hereafter referred to as distinct species). The plant specimens are deposited in the Herbarium of the Laboratory of Biogeography and Ecology of the University of the Aegean.

### 2.4 Functional traits

To assess how fire regime (viz. burnt vs. unburnt) impacts the functional composition of hoverfly communities, we considered five functional traits concerning either the larval or the adult stages (details are presented in Table S2):

- Number of generations per year (viz., univoltine, bivoltine, and multivoltine).
- Larval diet (viz., herbivore, carnivore, omnivore, and microphage).
- Larval habitat (viz., aquatic and terrestrial).
- Larval terrestrial habitat (viz., strictly aboveground, above‐ and belowground).
- Migratory status of the adults (viz., migratory and non‐migratory).

All functional trait data derived from existing literature are included in the *Syrph the Net* (*StN) Database* (Speight & Castella, 2020; Speight et al., 2020).

For the analysis, we selected the traits that are important for hoverfly ecology. Since StN Database includes data for all of Europe, we selected categorical functional traits that are valid for the Mediterranean (e.g., we excluded phenology data) and may be relevant to hypotheses regarding fire survival (viz., larval habitat and larval terrestrial habitat) and advantages in the post‐fire landscape (viz., number of generations per year, larval diet, and migratory status). Each hoverfly species was described in terms of the above five functional traits (Table S3), except for 11 specimens that were identified as three different morphospecies and were excluded from the functional trait analysis. Five specimens belonging to the species complex *Chrysotoxum intermedium* were also excluded from this analysis due to unresolved taxonomy (M.C.D. Speight, personal communication).

### 2.5 Statistical analysis

#### 2.5.1 Hoverfly abundance and diversity

We pooled data from the pan‐traps and hand‐netting surveys to calculate insect abundance (i.e., the number of individuals per species collected at each site), species richness (i.e., the number of species collected at each site), and *Shannon–Wiener diversity index (H)* (i.e., a widely used information statistic index, estimating the uncertainty of the next species to be found in a community, taking into consideration the abundance of species in a sample). Combining species data from pan‐traps and hand‐netting surveys were possible because both methods were standardized with equal effort at all sites and in all years (see also Lázaro, Tscheulin, Devalez, Nakas, & Petanidou, 2016; Minachilis et al., 2020; Lázaro et al., 2021). To estimate if hoverfly abundance is significantly predicted by fire regime in each post‐fire year, we applied generalized linear mixed‐effects (GLME) models (Poisson family). Due to detected overdispersion, we corrected the standard errors using a quasi‐GLM model where the variance is given by φ × μ, where μ is mean and φ the dispersion parameter. The response variable was the number of hoverfly individuals collected in each site. We used the site as a random factor and as explanatory variables the fire regime of the site (burnt and unburnt), together with three metrics representing plant diversity [viz., Shannon diversity of plants, number of species of plants, and the proportion (%) of hoverfly host‐plants floral abundance to the total floral abundance in each site]. We tested for collinearity among the latter three predictors and found that r_max_ = |0.481| and r_mean_ = |0.391|, thus we used all three of them. For the best‐fitted model, a stepwise backward selection procedure was applied using the Akaike information criterion. We should note that the Poisson GLME models used were not overdispersed because the ratio between the residual deviance to residual degrees of freedom was <1.1 (Zuur et al., 2009). For this analysis, we used the function *glmer()* of the R package lme4 v. 1.1–27.1.

To test if hoverfly species richness was correlated with fire regime (burnt vs. unburnt sites) across the post‐fire years, we used Poisson GLME models (which were not overdispersed), with species richness as response and fire regime as explanatory variables, using site as a random factor. For the case of the Shannon index, we used Gaussian LME models, with site as a random factor.

#### 2.5.2 Species functional traits

To test if fire regime predicts the abundance of individuals with different functional traits (number of generations, migration ability, and larval food resources and activity zones), we used quasi‐Poisson models, as above [quasi‐Poisson family in the R function *glm()*] (Zuur et al., 2009). The abundance of each trait category was used as response variable (using data from both pan‐traps and hand netting), and fire regime (viz., burnt and unburnt) as explanatory variable. The same models were applied to test the effect of the site category (viz., core, perimeter, and unburnt) on the population size of the migratory species.

#### 2.5.3 Species composition

To test for differences in the quantitative composition of hoverfly communities (using data from both pan‐traps and hand netting) in the sites of the different site categories (core, perimeter, and unburnt sites), we applied multivariate‐response generalized Linear mixed models (MGLMs). This approach fits a generalized linear model to the total community composition matrix, using a common *n*‐dimensional set of explanatory variables and a resampling‐based hypothesis testing (Wang et al., 2012). We built an MGLM model using the function *manyglm()* (family: negative binomial) of the R package mvabund v.4.1.12, with site category as the explanatory factor. The statistical significance of the fitted models was assessed with ANOVA (likelihood ratio tests) using 999 bootstrap iterations via PIT‐trap residual resampling. Univariate post hoc tests were then performed to determine which species varied significantly in their distribution among the different site categories.

To visualize the differences in the species' quantitative composition among the communities in the three site categories, we applied non‐metric multidimensional scaling (NMDS) with Bray–Curtis distance using the function *metaMDS()* in the R package vegan v.2.5–7.

#### 2.5.4 Host plants

In each one of the nine plant–hoverfly networks (see section 2.5.5), we calculated the phylogenetic diversity of the host‐plant assemblages. Faith's phylogenetic diversity (PD) measures phylogenetic richness, expressing the total amount of evolutionary history in the assemblages. This metric quantifies how rich are the hoverfly host communities in phylogenetic clades in the three site categories (see also Tucker et al., 2017).

The phylogenies of the plant assemblages that were visited by hoverflies in each site were built using the online software Phylomatic v.3 (tree R20120829) (Webb & Donoghue, 2005). We used the “bladj” algorithm in the software Phylocom v.4.2 (Webb et al., 2008) in order to adjust branch lengths of the phylogeny to correspond to evolutionary divergence time between clades, using the node ages by Gastauer & Alves Meira‐Neto (Gastauer & Alves Meira‐Neto, 2013). The indices of phylogenetic diversity were calculated with the function *pez.shape()* in the R package pez v.1.2–3.

#### 2.5.5 Plant–hoverfly meta‐networks

Here, we aimed at monitoring changes in the patterns of hoverfly–plant interactions across fire regimes and post‐fire years. For this analysis, only the hand‐netting data were used. We combined the networks in the sites at the level of regime category, resulting in three meta‐networks for each post‐fire year (i.e., core, perimeter, and unburnt); this was useful because of the small size of the hoverfly–plant networks at the site level (see also Librán‐Embid et al., 2021; Medel et al., 2022, for similar applications). Consequently, our point of reference has been the regime category at the regional (island) level (Table 4). Given the similar sampling efforts and the uniform pre‐fire ecosystem type across sampling sites across regime categories, we deem this upscaling acceptable for addressing our research question.

Given this caveat, we calculated four basic network‐level metrics which provide information about the post‐disturbance structure of the plant–hoverfly interacting communities and their susceptibility over time against subsequent disturbance (e.g., grazing):

- *Modularity* according to Beckett's algorithm (Beckett, 2016) using the quantitative version of the interactions’ matrix. This metric indicates the degree to which species tend to interact more strongly within a specific group of partners than with the rest of the network. Modularity was used because it is a useful indicator of the potential speed of the spread of a disturbance within an interacting community (Olesen et al., 2007; Ramos‐Robles et al., 2018; Sheykhali et al., 2020; Liu et al., 2021).
- *Nestedness:* the metric NODF was used, which describes the asymmetry of the network, i.e., the degree to which specialist insects tend to interact with subsets of the plants with which generalist insects interact and vice versa. Representing a measure of the asymmetry in trophic specialization across the community, nestedness is an inherent attribute of bipartite networks and may be linked with the post‐disturbance dynamics and the robustness of a community (Mariani et al., 2019).
- *Specialization* (H2′ index): a network‐level metric describing the extent to which the observed interactions deviate from a random selection of partners, taking into account the strength of each link, i.e., the frequency of each plant–pollinator interaction (Blüthgen et al., 2006). Post‐disturbance trophic specialization may predict susceptibility to subsequent disturbance (e.g., local extinctions of species due to post‐fire grazing) (Jordano et al., 2006; Forister et al., 2012; Schleuning et al., 2012).
- *Connectance*: a simple network metric expressing the proportion of realized interactions against the total possible interactions within the community. It is considered a measure of complexity and robustness of post‐disturbance interacting communities (Dunne et al., 2002a, 2002b)

For calculating the network metrics, we used the relative functions in the R package bipartite v.2.16. To address biases and facilitate comparison between networks, we used a null model approach for NODF, H2’, and connectance (Adedoja et al., 2019). Specifically, for these metrics, we additionally estimated their values based on 1000 networks generated by a null model [model “r2dtable”; function *nullmodel()*], and calculated the *z*‐scores between the observed and the estimated values.

For each plant species in each network, we calculated their contribution to the nested structure, by applying the function *nestedcontribution()* in the bipartite package. This metric predicts the degree to which the removal of these species will cause the collapse of the entire network (Saavedra et al., 2011), thus we used it to identify the plant species that are pivotal for the stability of the plant–hoverfly communities across years and fire regimes. For each focal plant species, this function compares its observed nestedness to the values calculated in a set of randomized interactions according to a null model and, finally, yields the *z*‐score from this comparison as the value of nestedness contribution. Positive values indicate species that increase the network's nestedness, while negative values species that decrease overall nestedness (see Saavedra et al., 2011).

## 3 RESULTS

### 3.1 Hoverfly abundance and diversity

Overall, we collected 910 individual hoverflies (542 by hand netting and 368 by pan‐traps), representing 19 genera and 49 species (among them three morphospecies; 34 collected with hand‐net, and 35 with pan‐traps). The most abundant species were *Sphaerophoria scripta* (128 individuals), *Merodon albifrons* (103), *Syritta pipiens* (92), *Eupeodes corollae* (77), and *Merodon neonanus* (70) (Table S4).

**FIGURE 2:**
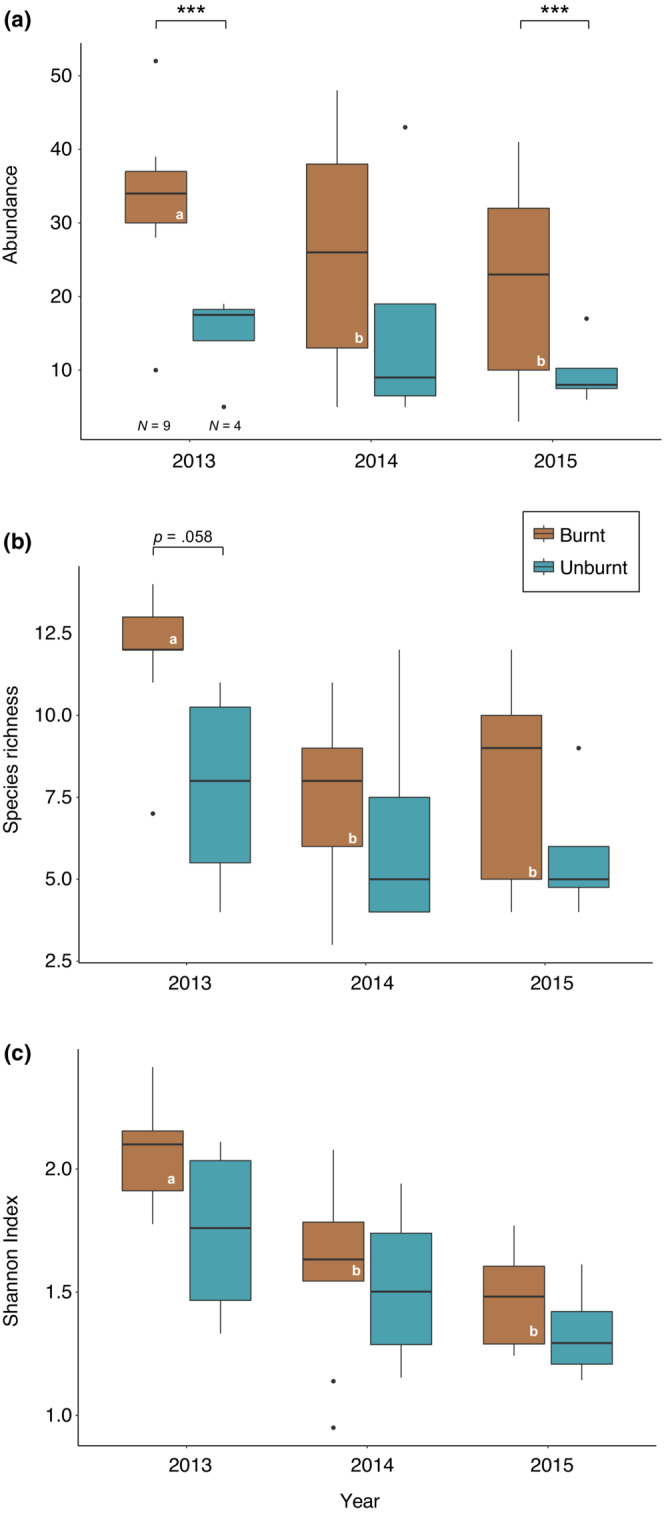
Comparison of hoverfly (a) abundance, (b) species richness, and (c) Shannon–Wiener diversity index between burnt and unburnt sites over 3 post‐fire years. Significant differences between burnt and unburnt sites within years are indicated by asterisks. Significant differences across years for sites of each category are indicated by letters. Results are based on Poisson GLM models. ***: *p* < .001; **: *p* < .01. Sample sizes are invariant across years.

Hoverfly abundance was higher in burnt sites compared with unburnt ones across all post‐fire years, although this difference was not significant in year 2 (Figure 2a). Fire regime (burnt vs. unburnt) was the most important predictor of the hoverfly abundance during year 1, according to the GLME models, followed by plant species richness (plant Shannon–Wiener index is not deemed significant because of the second best‐fitted model) (Table 1, Figure 2a). In year 2, the best predictor for hoverfly abundance was plant species richness; in year 3, hoverfly abundance was correlated with two of the metrics used: fire regime and the proportion of recorded hoverfly host plants in the flower abundance of the sites (Table 1). Hoverfly species richness (Figure 2b) and Shannon–Wiener index (Figure 2c) were also higher for burnt sites across all 3 post‐fire years, although the differences were not statistically significant.

**TABLE 1:** Predictors of hoverfly abundance during the three first post‐fire years: (i) fire regime (burnt vs. unburnt sites), (ii) plant Shannon index (H plants), (iii) plant species richness (S plants), and (iv) the proportion (%) of hoverfly host‐plants floral abundance to the total floral abundance in each site (% Abundance).

Post‐fire year		|ΔAIC|	*χ* ^2^	*p*	*β*‐Coefficients			
					Fire regime	H plants	S plants	% Abundance
2013	Model 1	9.75	15.75	.001	−0.95 ± 0.09	−0.16 ± 0.09	0.26 ± 0.09	−
	Model 2	9.23	13.23	.001	−0.95 ± 0.09	−	0.22 ± 0.09	−
2014	Model 1	10.93	14.94	.019	−	−	0.41 ± 0.21	−0.32 ± 0.21
	Model 2	10.42	13.23	.001	−	−	0.68 ± 0.15	
2015	Model 1	4.05	10.05	.018	−0.89 ± 0.34	0.36 ± 0.19	−	0.59 ± 0.19
	Model 2	2.90	6.91	.032	−1.05 ± 0.38	−	−	0.42 ± 0.19

*Note*: For each comparison, the results of the two best‐fitted GLME models (Model 1 &2) according to AIC criterion are shown. ΔAIC represents the AIC difference of the respective model from the null model (two models are generally equivalent if |ΔAIC| < 2).

In year 1, hoverfly communities in burnt sites had more individuals (Figure 2a) and exhibited greater species richness (GLME: *χ* ^2^ = 11.15, *p* = .004) (Figure 2b) and diversity (Shannon index, LME: *F*_*2,24*_ = 13.06*, p* < .001) (Figure 2c) compared with communities at the same sites in the following years; no significant differences were observed for these communities between years 2 and 3. Despite the decreasing trend, there were no significant differences across years for unburnt communities (Figure 2).

### 3.2 Species functional traits

In year 1, burnt communities had significantly more species having larvae that are as follows: (a) carnivorous, (b) non‐aquatic, or (c) dwelling strictly aboveground, compared with unburnt communities in the same year or with burnt communities in the following years (Table 2). Burnt communities in year 1 also had significantly more opportunistic species, which are migratory or have >2 generations per year, compared with unburnt communities in the same year (Table 2). All these differences disappeared during year 2. Finally, the burnt communities of year 3 had a higher abundance of species having larvae that are as follows: (a) microphage, (b) aquatic, or (c) dwelling belowground, compared with the unburnt communities of the same year; in contrast, they had a lower abundance of species with carnivorous larvae (Table 2). The comparison of the burnt communities across years revealed that the first 2 years had significantly more species having larvae that are (a) carnivorous, (b) non‐aquatic, and/or (c) dwelling strictly aboveground and species that are migratory or have >2 generations per year, compared with the communities of year 3. There were no significant differences across years for unburnt communities (Table 2).

**TABLE 2:** Comparative effects of fire on species functional traits between burnt and unburnt sites during the three first post‐fire years.

Comparison			Voltinism	Larval diet		Aquatic larvae		Terrestrial larvae		Migratory	
			Multivoltine	Microphages	Carnivores	Yes	No	Aboveground	Above‐ and/or belowground	Yes	No
Burnt–Unburnt within years	1st B	1st U	B > U***	−	B > U***	−	B > U***	B > U***	−	B > U***	−
	2nd B	2nd U	−	−	−	−	−	−	−	−	−
	3rd B	3rd U	−	B > U*	U > B*	B > U*	−	−	B > U*	−	B > U**
Burnt across years	1st B	2nd B	1st >2nd*	−	−	−	1st >2nd*	−	−	−	−
	1st B	3rd B	1st >3rd*	−	1st > 3rd***	−	1st >3rd*	1st >3rd***	−	1st >3rd**	−
	2nd B	3rd B	2nd >3rd*	−	2nd >3rd***	−	2nd >3rd*	2nd >3rd***	−	2nd >3rd**	−
Unburnt across years	1st U	2nd U	−	−	−	−	−	−	−	−	−
	1st U	3rd U	−	−	−	−	−	−	−	−	−
	2nd U	3rd U	−	−	−	−	−	−	−	−	−

*Note*: Only trait groups with statistically significant effects are shown (1st B: year 1, burnt communities; 1st U: year 1, unburnt communities; 2nd B: year 2, burnt communities; 2nd U: year 2, unburnt communities; 3rd B: year 3, burnt communities; and 3rd U: year 3, unburnt communities). The results shown are according to GLMs (Poisson distribution). ***: *p* < .001; **: *p* < .01; *: *p* < .05; and −: ns.

**FIGURE 3:**
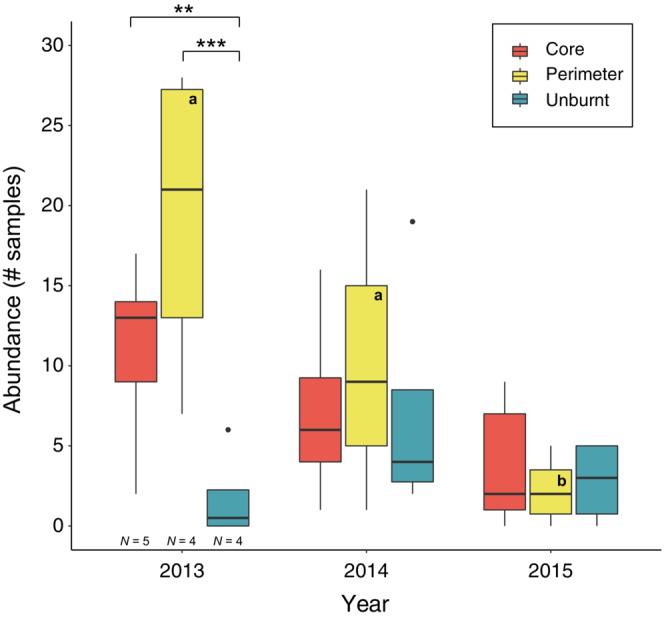
Comparison of the abundance of migratory hoverfly species between core‐burnt, perimeter‐burnt, and unburnt study sites in each post‐fire year. Significant differences between site categories within years are indicated by asterisks. Significant differences across years for sites of each category are indicated by letters. Results are based on quasi‐Poisson GLM models and post hoc tests. ***: *p* < .001; **: *p* < .01. Sample sizes are invariant across years.

The analysis considering only migratory species revealed that burnt sites (both core and perimeter) had significantly higher migratory hoverfly populations compared with unburnt sites in year 1 (Figure 3). Perimeter sites also had higher populations of migratory species compared with core sites during years 1 and 2 (Figure 3), although these differences were not statistically significant.

### 3.3 Species composition

**FIGURE 4:**
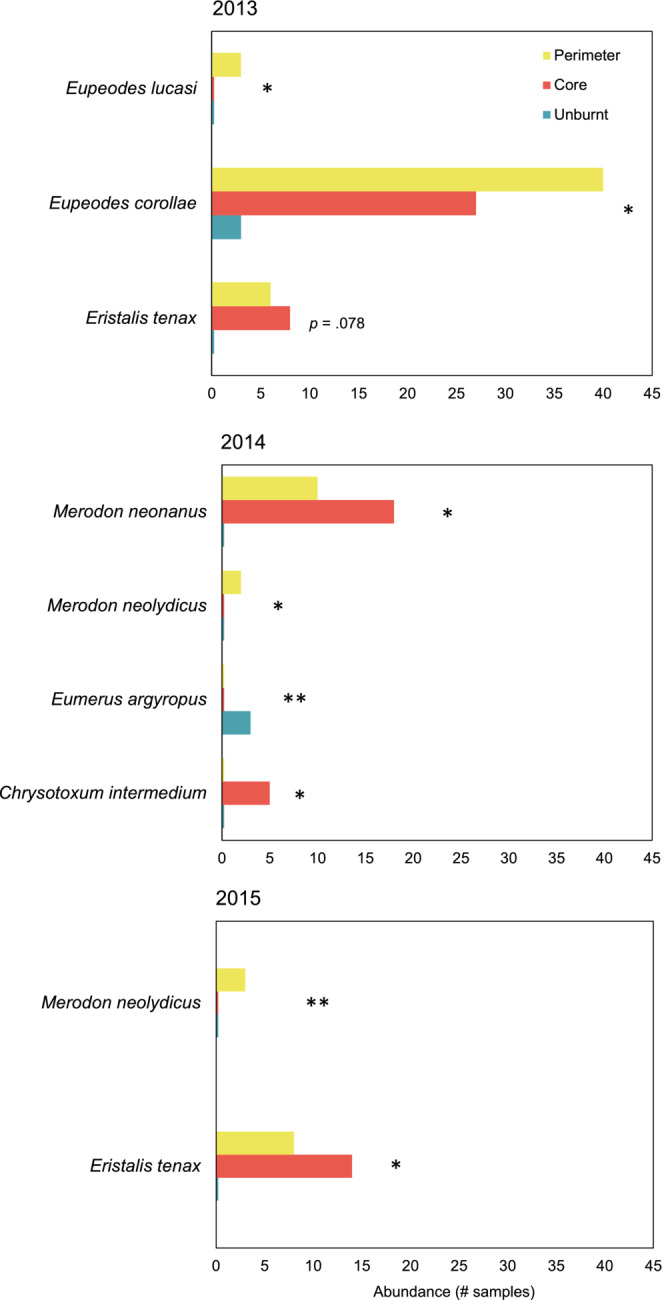
Hoverfly species showing a significant response to fire during the first 3 post‐fire years. Results refer to the univariate models of the MGLM analysis. **: *p* < .01; *: *p* < .05.

The MGLM analysis highlighted three species (*Eristalis tenax*, *E. corollae,* and *Eupeodes lucasi*) showing significant responses to fire in year 1 (Figure 4). All these species are migratory and almost absent from the unburnt sites during that year; the two *Eupeodes* spp. were most abundant in the perimeter sites, whereas *E. tenax* was more abundant in the core sites. Four non‐migratory species responded to fire in year 2 with *Eumerus argyropus* being more abundant in the unburnt site, *Merodon neolydicus* in the sites of the perimeter, and *Chrysotoxum intermedium* and *Merodon neonanus* in the core sites (Figure 4). Finally, two species responded significantly in year 3: *M. neolydicus* being more abundant in the sites of the perimeter and *E. tenax* in the core sites (Figure 4).

**FIGURE 5:**
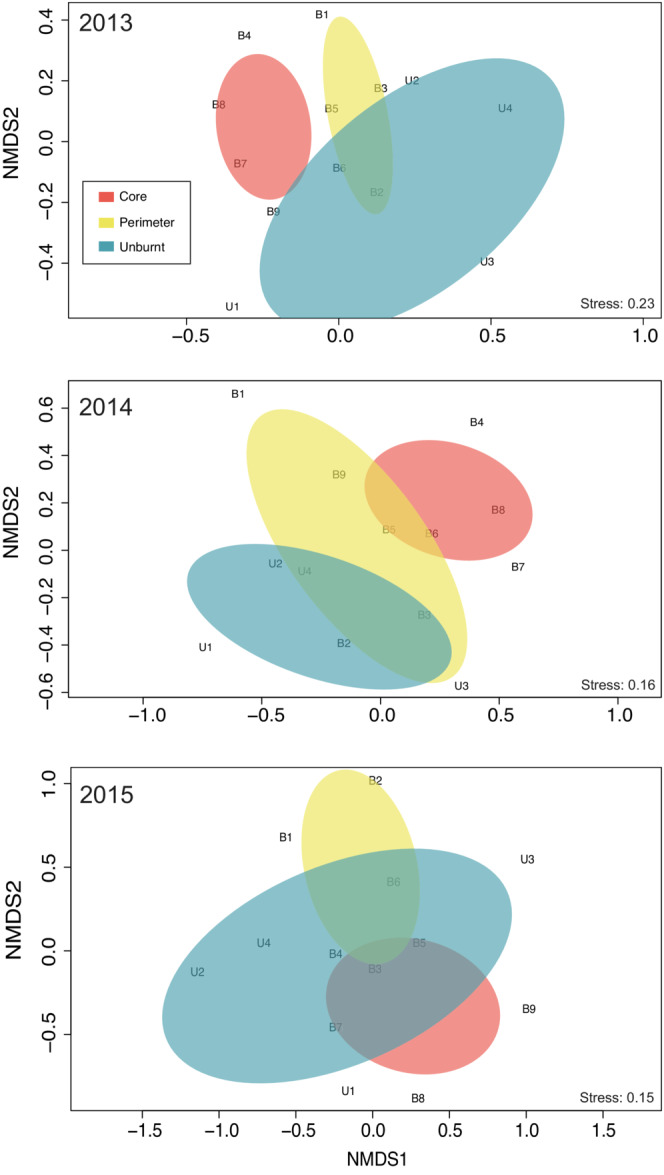
Non‐metric multi‐dimensional scaling (NMDS) plots based on Bray–Curtis distances for the hoverfly community composition in the sites among the 3 post‐fire years.

During year 1, the core sites had the most distinct communities compared with the communities from the two other categories of sites that partially overlap (Figure 5). These dissimilarities become less obvious during year 2 and disappear in year 3 (Figure 5).

### 3.4 Host plants

In total, hoverflies visited 66 flowering plant species belonging to 55 genera and 21 families. The most species‐rich family was Asteraceae (15 species), followed by Apiaceae (nine species) and Lamiaceae (eight species) (Table S5). The most visited plant species in burnt sites were herbaceous species like *Bupleurum gracile, Crepis commutata,* and *Hirschfeldia incana*; in the unburnt sites, the shrub *Origanum onites* was the main hoverfly pollinator host. Data about the nestedness contribution of each plant species in each network are presented in Table S6 in the Supporting Information.

Hoverflies visited more plant species in the core sites than in any other site category across the 3 years, with the unburnt sites having the lowest host‐plant diversity (Table 3). Dendrograms of the relevant plant assemblages revealed differences considering the diversity of host‐plant species among fire regimes and site categories (Figure S1). In the burnt sites, we found that host‐plant communities of either core or perimeter were more phylogenetically diverse and with higher values of Faith's PD compared with the unburnt sites (Table 3). Finally, within each of the two burnt categories (core or perimeter), Faith's PD declined with post‐fire time; in contrast, PD values remained relatively constant across years in the unburnt sites.

**TABLE 3:** Most‐visited plant species, Faith's phylogenetic diversity (PD), and number of plant species, genera, and families visited by hoverflies during the 3 first post‐fire years. For details on PD, see Materials and Methods.

	Year	# species	# genera	# families	Most visited species	PD
Core	2013	24	24	9	*Bupleurum gracile*	1793.00
	2014	21	20	7	*Crepis commutata*	1491.23
	2015	14	14	6	*Cistus creticus*	1339.93
Perimeter	2013	16	16	9	*Hirschfeldia incana*	1539.32
	2014	19	16	8	*Crepis commutata*	1418.47
	2015	8	6	5	*Helichrysum stoechas*	937.17
Unburnt	2013	11	10	5	*Origanum onites*	1058.82
	2014	13	12	5	*Origanum onites*	1144.67
	2015	13	12	7	*Origanum onites*	1254.33

### 3.5 Plant–hoverfly meta‐networks

Across all sites and years, we observed 542 interactions between 34 hoverfly species and 66 plant species in flower. Hoverflies interacted most frequently with *Daucus carota* (Apiaceae), *C. commutata* (Asteraceae), *Cistus creticus* (Cistaceae), *Helichrysum stoechas* ssp*. barrelieri* (Asteraceae), and *H. incana* (Brassicaceae) (Table 3; Table S5).

**FIGURE 6:**
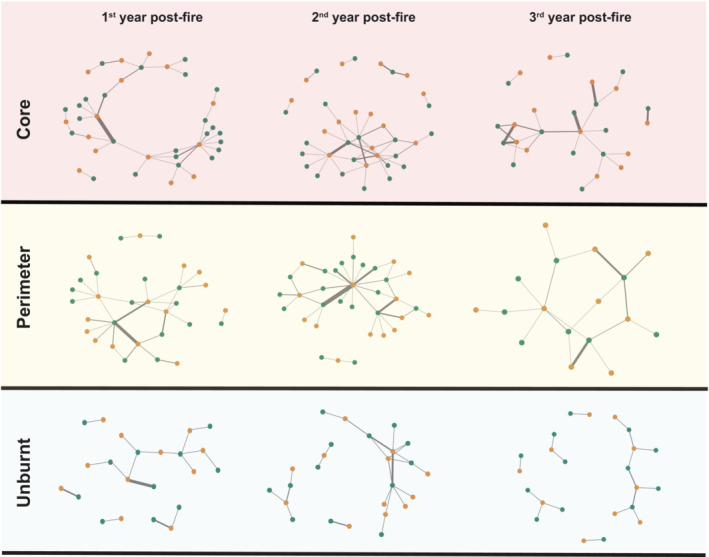
Meta‐networks of plant–hoverfly interactions during the 3 first post‐fire years in the three site categories. Green dots indicate plants and brown dots hoverflies. Bar thickness indicates the intensity (observed frequency) of interactions between each pair of species.

The largest meta‐networks were observed in the core sites during the first two post‐fire years and the smallest at the perimeter sites during year 3 (Table 4, Figure 6). In general, and with a few exceptions, burnt sites of both core and perimeter were more nested, less modular, and less specialized, compared with the unburnt sites (Table 4).

**TABLE 4:** Network metrics during the 3 first post‐fire years.

	Year	Average network size per site	Modularity	# modules	Nestedness (NODF)	Specialization (H2’)	Connectance
Core	2013	72.0	0.642	6	3.628	−5.666	5.802
	2014	67.2	0.560	10	2.823	−8.021	5.809
	2015	36.4	0.622	6	5.137	−14.686	9.979
Perimeter	2013	60.0	0.489	6	3.240	−6.455	3.193
	2014	71.3	0.497	6	3.226	−8.619	4.850
	2015	18.0	0.522	3	2.106	−6.439	4.149
Unburnt	2013	30.3	0.713	8	2.096	−4.340	3.147
	2014	39.0	0.507	7	3.434	−6.566	5.359
	2015	32.5	0.691	7	3.226	−4.655	3.791

*Note*: For NODF, H2’, and connectance, the z‐scores based on the comparison with the values calculated in 1000 null models are reported. For details on the metrics, see Materials and Methods.

## 4 DISCUSSION

To our knowledge, this study is the first to document responses of interacting plant–hoverfly communities to fire. By integrating biodiversity assessments, hoverfly functional traits of both adults and larvae, as well as network analysis, we disclosed the post‐fire timeline of events toward the recovery of a Mediterranean community during the first 3 post‐fire years. We observed larger and more diverse populations of hoverflies in the burnt sites during year 1. Furthermore, fire seems to favor migratory and/or multivoltine species, which were found to establish large populations, especially in the first post‐fire flowering season. Finally, the post‐fire plant–hoverfly networks were initially large and generalized, gradually becoming more compact. Each of these findings has important implications for ecology and conservation in the fire‐prone Mediterranean‐type ecosystems, as discussed in detail in the following sections.

### 4.1 Fire effects on hoverfly abundance and diversity

In studies monitoring biodiversity change, it is important to consider species abundance along with richness because changes in abundance constitute the most critical step preceding species turnover (Jandt et al., 2022). In our case study, fire has had a significant impact on hoverfly abundance, with burnt sites encompassing larger populations compared with the unburnt ones (Figure 2, Table 1). The only research findings comparing post‐fire hoverfly populations with unburnt ones are from a boreal forest in Sweden, reporting no significant changes in hoverfly abundance, however, only for the third post‐fire year (Johansson et al., 2020). This contrast might reflect differences in prevailing environmental conditions and species composition between the boreal and the Mediterranean environments.

Many insects, including pollinators, can be fire dependent (i.e., species that require fires for survival, albeit without showing any particular morphological adaptations), and some may even benefit from resulting ecosystem changes in the early successional stages (Lazarina et al., 2019; Pausas & Parr, 2018). For instance, bee abundance can be enhanced by changes in the vegetation structure with decreased forested habitat and increased availability of floral resources in early post‐fire landscapes (Burkle et al., 2019; Potts et al., 2003). In the current study, floral density and diversity partly explained the increased hoverfly abundance in the burnt sites (Table 1), suggesting that fire may indirectly affect insects in various ways, including the increased availability of nesting habitats, as previously shown for bees (Burkle et al., 2019), as well as changes in habitat type. The latter is known to be pivotal for hoverflies (Miličić et al., 2021; Speight et al., 2020) and it is highly relevant to our system, as most of our burnt sites were fire transformed from forests and maquis habitats into grasslands with only few remaining trees and shrubs.

Hoverfly species richness and diversity were higher in burnt sites in year 1 compared with the unburnt ones, and in the following years, again suggesting that some species moved away from the burnt sites after the first flowering season. Higher species diversity in burnt areas has been observed for also other insect pollinator guilds, especially bees (e.g., Burkle et al., 2019; Moretti et al., 2009; Potts et al., 2003).

### 4.2 Fire effects on species functional traits

Fire regimes were differentially associated with hoverfly functional traits (Table 2). Considering the larval activity zone, our initial hypothesis was that some species would be favored because their larvae could be protected from fire (besides, larvae are less mobile than adults). However, while we had expected to find larger populations of species with belowground or aquatic larvae in burnt vs. unburnt sites, we found larger populations of species that nest strictly aboveground and their larvae do not have an aquatic larval phase (Table 2). A possible explanation is that the recorded species did not survive the fire as larvae, but mostly as adults and/or have moved to the burnt areas from the nearby unburnt ones. Many species have long‐flying periods, with active adults during the summer and large populations even during autumn (Petanidou et al., 2011; Speight et al., 2020). Most of the species in our sites (37 of the 45 species used in the analysis) are known to have flight periods that, in Greece, include or exceed August (Vujić et al., 2020), the month the fire incident occurred (18–28 August 2012). The majority of these species (62% in our dataset) have >2 generations per year (Table S3), thus the chances of them being adults at the time of the fire incident were very high. Thus, we hypothesize that the recorded differences in larval traits are probably a result of recolonization from the unburnt areas in combination with reduced adult mortality mostly of multivoltine species; in fact, multivoltine species are known to be opportunistic, usually producing many eggs (Zheng et al., 2019) and being able to readily exploit the post‐fire conditions even from the first weeks as we observed in November 2012.

We found strong effects on larval feeding habits, with larger populations of carnivores in the burnt sites during year 1, decreasing significantly with time (Table 2). These carnivorous species mainly feed on aphids that live in all different types of vegetation (Day et al., 2015; Vujić et al., 2020). Aphid populations are strongly affected by fire events, and aphids can exhibit reduced population numbers for long periods in the post‐fire years, as some species are less mobile and may require more time to recolonize the burnt area than many other insects (Harper et al., 2000). Apparently, many of the carnivorous aphid consumers survived as adults the fire and foraged in the freshly burnt sites, with their population radically reduced during the next years (Table 2), possibly following the reduced populations of aphids. Our results suggest opposite trends between carnivorous and microphagous larvae: by year 3, burnt sites had fewer carnivorous species compared with the unburnt ones, which was reverse for the microphages (Table 2). Most of these microphages are saproxylic (Vujić et al., 2020), and possibly they were favored in and/or moved to the burnt sites owing to the newly available resources of dead wood.

As expected, burnt sites had larger population sizes of migratory species, but not of non‐migratory species (Table 2). Earlier studies on butterflies also showed that recently burnt areas have larger populations of widely dispersed species compared with the less widely dispersed ones (Swengel, 1996). Cosmopolitan migratory hoverflies (e.g., *E. tenax*, *Episyrphus balteatus,* and *E. corollae*) are known to migrate every year for very long distances (Jia et al., 2022; Pérez‐Bañón et al., 2003, 2007; Wotton et al., 2019). We speculate that several migratory species in the burnt sites were newcomers that moved in from adjacent unburnt areas, considering that post‐fire sites are ideal habitats for these opportunistic species as soon as flowers are available (Carbone et al., 2019). Furthermore, fire is known to result in a lack of post‐fire landscape connectivity (Burgio et al., 2015) and the destruction of ecological corridors in the burnt areas (Burgio et al., 2015; Nabhan, 2004). This destruction may have prevented less capable dispersers to forage in the core sites and resulted in larger populations (although statistically non‐significant) confined in the perimeter sites (Figure 3). In consequent post‐fire years, the post‐fire hoverfly populations of migratory species were reduced, probably due to competition with less opportunistic, k‐strategist species.

### 4.3 Fire and hoverfly species composition

The MGLM and NMDS analyses revealed that the differences in species composition are larger when comparing core burnt sites with both the unburnt sites and the burnt sites of the perimeter (Figures 4 and 5). These dissimilarities may reflect species turnover in burnt sites compared with the unburnt ones, and changes in the relative abundance in core sites compared with the perimeter ones. Our results showed that in year 1, the observed differences were driven by *E. tenax*, *E. corollae,* and *E. lucasi* (Figure 4). These three species are migratory, forming larger populations in the burnt sites compared with the unburnt ones where they were almost absent (Figure 4, Table S4). The two *Eupeodes* species are medium sized (Speight et al., 2020), and they formed larger populations in the sites of the perimeter. *E. tenax* is larger (Speight et al., 2020) and was more abundant in the core sites. We speculate that, in addition to the populations that survived in the burnt sites, insects from all three migratory species moved from elsewhere to exploit the freshly available food sources; the differential presence of *E. tenax* in the core sites may be explained by its better ability to disperse (Rader et al., 2011).

The core sites were not only distinct from the two other site categories but also clustered together, indicating higher similarity in species composition (Figure 5). This is consistent with previous studies showing that fires can function as filters, shaping communities with different species compositions compared with the unburnt ones (Johansson et al., 2020; Lazarina et al., 2017). Differences in species composition and community structure became less intense during year 2 (in which non‐migratory species drive the dissimilarities observed), and almost disappeared during year 3 (Figures 4 and 5).

### 4.4 Fire and host plants

Fire may also affect hoverflies via effects on the plant communities with which they associate. Most hoverflies are generalist foragers (Larson et al., 2001): in China, Jia et al. (2022) showed that the migratory species *E. balteatus* can feed, all‐in‐all, on >1000 plant species. Potts et al. (2003) reported reduced levels of floral food resources (in terms of pollen and nectar energy) in a burnt pine forest in Israel during year 1, even though the overall plant diversity was high. In such conditions, generalist opportunistic species might be expected to exploit any available food resources (Branquart & Hemptinne, 2000). This is consistent with our results (Table 3) showing that hoverflies foraged on 24 plant species in the core sites of year 1, compared to 11 plant species of the unburnt sites (over a total of 86 and 71 plant species in flower, respectively), a trend also reflected in the higher values of Faith's phylogenetic diversity (PD) in the burnt sites (Figure S1) (see also Tucker et al., 2017). Considering the temporal trend, we observed an important decline from year 1 to year 3, both in the core and in the perimeter, which again is reflected in the number of visited plant species and the values of PD (Table 3).

Fires are known to change vegetation structure and diversity (Burkle et al., 2022; Moretti et al., 2009). Consistent with findings from previous studies (e.g., Moretti et al., 2009; Potts et al., 2003), our post‐fire plant communities were dominated by herbaceous plants (see Table 3 and Figure S1), with bowl‐shaped flowers including several members of Asteraceae and Apiaceae. While hoverflies have also been found to visit a wide range of flowers from several families (see Vujić et al., 2020 and refs. therein), they typically have short mouth parts (Gilbert, 1981) and tend to prefer bowl‐shaped flowers that provide easy access to nectar and pollen, as well as convenient platforms for landing and take‐off (Vujić et al., 2020). However, even short‐tongued species can visit long‐spurred flowers for pollen and lick nectar at the entrance of the spur (Vlašánková et al., 2017); this behavior was also observed in our communities (e.g., plants of the family Lamiaceae).

The two most important plants for sustaining hoverfly assemblages in our Mediterranean community are *C. commutata* and *D. carota,* which positively contributed to the nested structure across site categories and years (Table S6). Furthermore, *H. incana, Sonchus asper, Leontodon tuberosus,* and *Scorzonera elata* were the most important contributors present exclusively in burnt sites (Table S6) and might have acted as key species for the post‐fire recovery of hoverfly communities. In contrast, in the unburnt sites, which had fewer herbs and more shrubs, hoverflies visited most frequently plants from the family of Lamiaceae (Figure S1, Table S6); in these sites, *O. onites* was the most important contributor to nestedness (Table S6). Analyses of both ecological and social networks have shown that nestedness predicts network collapse after the removal of the focal nodes (Saavedra et al., 2011). Therefore, the species mentioned here may represent key plants for hoverfly conservation in the Mediterranean communities, and their removal due to some subsequent disturbance after the fire is expected to be detrimental to the community recovery.

### 4.5 Fire effects on plant–hoverfly networks

The meta‐networks in the burnt communities during year 1 were larger than those in unburnt communities (Figure 6, Table 4) involving more species of both insects and plants (Table 3). Petanidou et al. (2008) previously showed that interaction turnover among years in Mediterranean communities can be driven not only by species turnover but also by the species' flexibility to interact with new partners. In our case, hoverflies that colonized the burnt sites had the opportunity to exploit an increased post‐fire plant diversity and forage on more plant species, probably resulting in the observed larger meta‐networks showing lower specialization and higher connectance compared with the unburnt sites (Table 4). Hoverflies are known for their generalized foraging behavior, at the community (Klecka et al., 2018), species, and even the individual levels (Jia et al., 2022; Lucas et al., 2018). In a post‐disturbance floral market, such general strategies are expected to provide an advantage for the recovery of pollinator populations. Similar were the trends found in earlier studies. Peralta et al. (2017) found that freshly burnt sites have higher abundances of generalist than specialist bees. In Mediterranean‐type habitats of the Cape Region, Adedoja et al. (2019) found the lowest values of specialization in burnt sites compared both with unburnt sites outside the burnt area and with unburnt refuges inside the burnt area.

Nestedness is another important attribute of bipartite mutualistic networks that is positively linked with the robustness of the interacting assemblages, the reduction of interspecific competition, and the survival of the community during environmental unpredictability and disturbance (Bascompte, 2009; Saavedra et al., 2016; Mariani et al., 2019). In the current study, post‐fire meta‐networks in burnt sites exhibited higher nestedness compared with the unburnt ones during year 1. Perhaps the higher flower and hoverfly species richness observed here resulted in the higher nestedness (Table 4), suggesting that new interactions emerged as hoverfly species exploited most of the available resources (but see Welti & Joern, 2018).

The plant–hoverfly meta‐networks of the burnt sites exhibited lower modularity than the unburnt ones, with perimeter sites showing the lowest values of all (Table 4). In bipartite networks, modules are groups of species interacting more strongly with each other compared with the rest of the community (Bascompte, 2009). This confirms the rather weak structure of the perimeter meta‐networks which is also indicated by the low connectance (Table 4). A highly modular structure is thought to be ecologically important, indicating a slow rate of the spreading of a disturbance across the network (Liu et al., 2021; Olesen et al., 2007; Ramos‐Robles et al., 2018; Sheykhali et al., 2020). In this context, fire perimeter constitutes the most vulnerable area compared with the core (modular and well‐connected meta‐networks) and the unburnt areas (less connected, but highly modular), which has major implications for conservation, especially in the face of additional disturbances that can impact the post‐fire landscape (e.g., intense livestock grazing, plant introductions, etc.).

Several studies have reported that interactions among plants and pollinators do not remain constant through time with recorded year‐to‐year variations even in the network metrics (Dupont et al., 2009; Olesen et al., 2008; Petanidou et al., 2008). Such variations are also observable in our post‐fire communities, both in the burnt and the unburnt ones; apart from the variations, we also observe clear trends regarding the burnt sites in which networks become smaller in the post‐fire period (year 3 vs. year 1), core sites less specialized, and perimeter sites less nested (Table 4). Thus, our results show that even though the differences between burnt and unburnt hoverfly communities fade with post‐fire time, differences in some network metrics between burnt and unburnt sites are still evident in year 3. Consequently, it remains unclear if the first three post‐fire years are sufficient for the complete restoration of the hoverfly and plant communities and their networks; thus, longer‐term monitoring is deemed essential for a better understanding of the related fire effects.

## 5 CONCLUSIONS

Our findings reveal clear effects of fire on hoverfly populations, functional diversity, and networks with host plants over the first 3 years following a fire event. Hoverfly abundance and species richness peaked during the first post‐fire year, with the hoverflies that survived the fire and/or colonized the burnt landscape (e.g., migratory species as habitat opportunists) constituting its primary pollinators. The positive effects of fire on diversity and abundance faded over the next 2 years, with hoverfly burnt communities and their interaction networks with their host plants eventually becoming more similar to the unburnt ones. We hypothesize that the latter is primarily due to migratory species being replaced by other rather k‐strategists slowly shifting to the recovering burnt sites. We also found that hoverflies in recently burnt sites consistently rely on specific herbaceous plants, the removal of which, in the case of further post‐fire disturbances that are common in the Mediterranean (e.g., grazing; Petanidou & Ellis, 1996, Lázaro, Tscheulin, Devalez, Nakas, & Petanidou, 2016, Lázaro, Tscheulin, Devalez, Nakas, Stefanaki, et al., 2016), might be predicted to cause a collapse of the hoverfly assemblage.

Taken together, these findings provide new insight into fire ecology and pollination that may help the development of management strategies aimed at protecting pollination services in the fire aftermath. However, we stress that even though the first 3 years provide a clear timeline of the post‐fire recovery of the community, longer‐term monitoring of burnt communities is pivotal for capturing in detail the sequence of events, and for understanding in depth the response of natural communities to wildfires that are projected to become more frequent and more destructive in the future, even in relatively fire adapted biomes such as those in the Mediterranean.

## AUTHOR CONTRIBUTIONS

**Georgios Nakas:** Conceptualization (equal); data curation (lead); formal analysis (equal); investigation (lead); methodology (equal); project administration (equal); visualization (equal); writing – original draft (lead); writing – review and editing (lead). **Aphrodite Kantsa:** Conceptualization (supporting); data curation (equal); formal analysis (lead); methodology (equal); visualization (equal); writing – review and editing (supporting). **Ante Vujic:** Data curation (equal); writing – review and editing (supporting). **Mark Mescher:** Funding acquisition (supporting); writing – review and editing (supporting). **Consuelo M De Moraes:** Funding acquisition (supporting); supervision (supporting); writing – review and editing (supporting). **Theodora Petanidou:** Conceptualization (equal); data curation (supporting); formal analysis (supporting); funding acquisition (lead); investigation (supporting); methodology (equal); project administration (equal); resources (lead); supervision (lead); writing – review and editing (supporting).

## ACKNOWLEDGEMENTS

We acknowledge the support of the Forest Service of Chios Island for allowing us to carry out fieldwork in the freshly burnt area and for providing the fire perimeter data. We thank M. C. D. Speight for providing us with the data of Syrph the Net (StN) Database; A. Grković for insect identification; T. Tscheulin and J. Devalez for helping in the field and the lab, respectively; and M. K. Panourgia for her help in creating the study sites map. Data collection was funded by Project THALES: POL‐AEGIS (Grant number MIS 376737) that was co‐financed by the European Union (European Social Fund‐ESF) and Greek national funds, through the Operational Program “Education and Lifelong Learning” of the National Strategic Reference Framework (NSRF)‐Research Funding Program.

## Supporting information


Appendix S1.

## Data Availability

The data used for the analyses in this article are available in its supporting information and in Figshare at: https://doi.org/10.6084/m9.figshare.21937820.v1.
