# Supplementary material for: Recent fire in a Mediterranean ecosystem strengthens hoverfly populations and their interaction networks with plants
Source: Ecol Evol. 2023 Feb 7;13(2):e9803. doi: 10.1002/ece3.9803 (PMC9905663; doi:10.1002/ece3.9803)
Supplement: Supplementary file 1 — Appendix S1. [file ECE3-13-e9803-s001.pdf]

Table S1: Permanent study sites in Chios Island.

Site	Fire regime	Site category	Distance from the fire perimeter
B1	Burnt	Perimeter	<500 m
B2	Burnt	Perimeter	<500 m
B3	Burnt	Perimeter	<500 m
B4	Burnt	Core	>1000m
B5	Burnt	Core	900 m
B6	Burnt	Perimeter	<500 m
B7	Burnt	Core	>1000m
B8	Burnt	Core	>1000m
B9	Burnt	Core	>1000m
U1	Unburnt	Unburnt	-
U2	Unburnt	Unburnt	-
U3	Unburnt	Unburnt	-
U4	Unburnt	Unburnt	-

Table S2: Hoverfly functional traits and their categories used in the study. Traits for each species are according StN database (Speight and Castella 2020, Speight et al. 2020).

**Functional trait**	**Trait description**	**Trait category**	**Category description**
Number of generations per year	The number of successive life cycles completed by a species during a year	1 generation	univoltine species: the life cycle takes a year to complete
		2 generations	bivoltine species: two life cycles are completed during the course of a year (one generation being more protracted than the other, due to overwintering)
		>2 generations	polyvoltine species: the number of successive life cycles completed within a year is greater than 2
Larval diet	Larval food, categorised according to whether it is living animals or plants, or decomposing organic matter	microphage	species whose larvae feed on micro-organisms associated with various forms of decomposing organic matter
		herbivore	species whose larvae feed on the tissues of living, non-woody plants
		carnivore	species whose larvae are predatory
		omnivore	species whose larvae feed on more than one categories
Larval activity zone – Aquatic	The habitat strata and microsites within which the larvae occur includes an aquatic phase	yes	microsite features occurring within surface water or groundwater
		no	does not include an aquatic larval phase
Larval activity zone – Terrestrial	The habitat strata and microsites within which the larvae occur	above ground	larval activity zone on: plants, timber, ground surface debris, nests of social insects
		below ground	larval activity zone on: roots
Migratory status (adults)	Categorisation of the species according to the extent to which they are known to undertake long-distance movements	migrant	species known to undertake long-distance movements
		non migrant	species not known to migrate

Table S3: Functional traits per hoverfly species according to StN database (Speight and Castella 2020, Speight et al. 2020).

	Migratory status (adult)	Larval activity zone – Aquatic	Larval activity zone – Terrestrial	Diet (larvae)	Number of generations per year
Brachypalpus valgus (Panzer, 1798)	No	No	Above	Microphage	1
Episyrphus balteatus (De Geer, 1776)	Yes	No	Above	Carnivore	>2
Eristalinus aeneus (Scopoli, 1763)	No	yes		Microphage	>2
Eristalis arbustorum (Linnaeus, 1758)	Yes	yes	Above	Microphage	>2
Eristalis similis (Fallén, 1817)	No	yes	Above	Microphage	2
Eristalis tenax (Linnaeus, 1758)	Yes	yes	Above	Microphage	>2
Eumerus amoenus Loew, 1848	No	No	Both	Omnivore	>2
Eumerus argyropus Loew, 1848	No	No	Both	Omnivore	>2
Eumerus basalis Loew, 1848	No	No	Both	Omnivore	2
Eumerus crassus Grković, Vujić & Radenković in Grković et al., 2016	No	No	Both	Omnivore	2
Eumerus lucidus Loew, 1848	No	No	Both	Omnivore	2
Eumerus pulchellus Loew, 1848	No	No	Both	Omnivore	2
Eumerus pusillus Loew, 1848	No	No	Both	Omnivore	2
Eumerus truncatus Rondani, 1868	No	No	Both		2
Eupeodes corollae (Fabricius, 1794)	Yes	No	Above	Carnivore	>2
Eupeodes lucasi (Marcos-García & Láska, 1983)	Yes	No	Above	Carnivore	>2
Helophilus trivittatus (Fabricius, 1805)	Yes	yes		Microphage	2
Melanostoma mellinum (Linnaeus, 1758)	Yes	No	Both	Omnivore	>2
Meliscaeva auricollis (Meigen, 1822)	Yes	No	Above	Carnivore	>2
Merodon albifrons Meigen, 1822	No	No	Both	Herbivore	2
Merodon avidus (Rossi, 1790)	No	No	Both	Herbivore	2
Merodon clunipes Sack, 1913	No	No	Both	Herbivore	1
Merodon italicus Rondani, 1845	No	No	Both	Herbivore	1
Merodon neolydicus Vujić in Vujić et al., 2018	No	No	Both	Herbivore	1
Merodon neonanus Vujić & Taylor in Vujić et al., 2015	No	No	Both	Herbivore	2
Merodon nigritarsis Rondani, 1845	No	No	Both	Herbivore	1
Merodon spinitarsis Paramonov, 1929	No	No	Both	Herbivore	1
Merodon testaceus Sack, 1913	No	No	Both	Herbivore	1
Merodon velox Loew, 1869	No	No	Both	Herbivore	2
Myathropa florea (Linnaeus, 1758)	No	yes	Above	Microphage	>2
Neoascia podagrica (Fabricius, 1775)	No	yes	Above	Microphage	>2
Paragus bicolor (Fabricius, 1794)	No	No	Above	Carnivore	>2
Paragus haemorrhous Meigen, 1822	No	No	Above	Carnivore	>2
Paragus pecchiolii Rondani, 1857	No	No	Above	Carnivore	>2
Paragus quadrifasciatus Meigen, 1822	No	No	Above	Carnivore	>2
Paragus tibialis (Fallén, 1817)	No	No	Above	Carnivore	>2
Scaeva dignota (Rondani, 1857)	Yes	No	Above	Carnivore	2
Scaeva pyrastri (Linnaeus, 1758)	Yes	No	Above	Carnivore	>2
Sphaerophoria rueppelli (Wiedemann, 1830)	No	No	Above	Carnivore	>2
Sphaerophoria scripta (Linnaeus, 1758)	Yes	No	Above	Carnivore	>2
Syritta pipiens (Linnaeus, 1758)	No	yes	Both	Microphage	>2
Syrphus ribesii (Linnaeus, 1758)	Yes	No	Above	Carnivore	>2
Syrphus vitripennis Meigen, 1822	Yes	No	Above	Carnivore	>2
Volucella zonaria (Poda, 1761)	Yes	No	Both	Carnivore	1
Xanthogramma citrofasciatum aff.	No	No	Both	Carnivore	2

Table S4: Total abundance of hoverflies collected by both pan traps and hand-netting in burnt (9 sites) and unburnt (4 sites) sites during the three years of sampling.

**Species**	**Burnt (9 sites)**	**Unburnt (4 sites)**	**Total (13 sites)**
Brachypalpus valgus	1		1
Chrysotoxum intermedium	5		5
Episyrphus balteatus	14	3	17
Eristalinus aeneus	7	3	10
Eristalis arbustorum	3		3
Eristalis similis	5	1	6
Eristalis tenax	59	2	61
Eumerus amoenus	23	4	27
Eumerus argyropus	10	3	13
Eumerus basalis	39	13	52
Eumerus crassus	2	8	10
Eumerus lucidus	3	1	4
Eumerus pulchellus	3	1	4
Eumerus pusillus	9	2	11
Eumerus spec.	5		5
Eumerus truncatus	1		1
Eupeodes corollae	74	3	77
Eupeodes lucasi	3		3
Eupeodes spec.	5		5
Helophilus trivittatus	1	1	2
Melanostoma mellinum	11	4	15
Meliscaeva auricollis	1	1	2
Merodon albifrons	88	15	103
Merodon avidus	1		1
Merodon clunipes	1	1	2
Merodon italicus	1	2	3
Merodon neolydicus	9	1	10
Merodon neonanus	62	8	70
Merodon nigritarsis		1	1
Merodon spinitarsis	25	2	27
Merodon testaceus	1		1
Merodon velox	22	31	53
Myathropa florea		1	1
Neoascia podagrica	1		1
Paragus bicolor	8		8
Paragus haemorrhous	1		1
Paragus pecchiolii	1		1
Paragus quadrifasciatus	2	1	3
Paragus spec.	1		1
Paragus tibialis	37	3	40
Scaeva dignota	5	1	6
Scaeva pyrastri	14	2	16
Sphaerophoria rueppelli	1		1
Sphaerophoria scripta	98	30	128
Syritta pipiens	78	14	92
Syrphus ribesii	3		3

Species	Burnt (9 sites)	Unburnt (4 sites)	Total (13 sites)
Syrphus vitripennis	1		1
Volucella zonaria	1		1
Xanthogramma citrofasciatum aff.		1	1

Table S5: Number of hoverfly species and individuals collected per plant species during the three years of sampling.

Plant species	Family	# of species collected	# of insects collected
Allium exile Boiss. & Orph.	Alliaceae	1	2
Allium neapolitanum Cirillo	Alliaceae	1	1
Anagallis arvensis L.	Primulaceae	3	6
Anchusa undulata L.	Boraginaceae	1	1
Anthemis chia L.	Asteraceae	1	2
Anthemis cretica L.	Asteraceae	2	3
Anthyllis hermanniae L.	Fabaceae	3	4
Asphodelus ramosus L.	Asphodelaceae	1	1
Bellevalia trifoliata (Ten.) Kunth	Hyacinthaceae	1	1
Biscutella didyma L.	Brassicaceae	1	1
Bupleurum gracile d'Urv.	Apiaceae	5	29
Centaurea urvillei DC.	Asteraceae	1	1
Centaurium tenuiflorum (Hoffmanns. & Link) Fritsch	Gentianaceae	1	1
Cistus creticus L.	Cistaceae	11	47
Cistus parviflorus Lam.	Cistaceae	2	4
Cistus salviifolius L.	Cistaceae	3	4
Convolvulus althaeoides L.	Convolvulaceae	3	7
Crepis commutata (Spreng.) Greuter	Asteraceae	11	60
Crepis multiflora Sm.	Asteraceae	2	2
Daucus carota L.	Apiaceae	11	80
Eryngium campestre L.	Apiaceae	2	7
Euphorbia acanthothamnos Heldr. & Sart. ex Boiss.	Euphorbiaceae	2	3
Fumana arabica (L.) Spach	Cistaceae	3	5
Fumana thymifolia (L.) Spach	Cistaceae	2	2
Gagea graeca (L.) Irmsch.	Liliaceae	1	1
Genista acanthoclada DC.	Fabaceae	2	2
Geranium purpureum Vill.	Geraniaceae	1	1
Glebionis coronaria (L.) Cass. ex Spach	Asteraceae	1	1
Helichrysum stoechas subsp. barrelieri (Ten.) Nyman	Asteraceae	7	43
Hirschfeldia incana (L.) Lagr.-Foss.	Brassicaceae	7	40
Hypericum empetrifolium Willd.	Hypericaceae	1	2
Hypochaeris achyrophorus L.	Asteraceae	5	11
Lamium amplexicaule L.	Lamiaceae	1	1
Leontodon tuberosus L.	Asteraceae	4	13
Leopoldia comosa (L.) Parl.	Hyacinthaceae	1	1
Lotus ornithopodioides L.	Fabaceae	1	1
Muscari commutatum Guss.	Hyacinthaceae	1	1
Ononis reclinata L.	Fabaceae	1	1
Origanum onites L.	Lamiaceae	7	28
Ornithogalum montanum Cirillo	Hyacinthaceae	1	1
Pallenis spinosa (L.) Cass.	Asteraceae	2	2
Picris rhagadioloides (L.) Desf.	Asteraceae	3	10
Pimpinella cretica Poir.	Apiaceae	7	10
Pimpinella peregrina L.	Apiaceae	5	9
Podospermum canum C.A. Mey.	Asteraceae	1	1
Ruta chalepensis L.	Rutaceae	1	1
Salvia fruticosa Mill.	Lamiaceae	3	3
Salvia viridis L.	Lamiaceae	2	2
Satureja thymbra L.	Lamiaceae	2	2

Plant species	Family	# of species collected	# of insects collected
Scaligeria napiformis (Spreng.) Grande	Apiaceae	4	9
Scandix pecten-veneris L.	Apiaceae	1	1
Scorzonera elata Boiss.	Asteraceae	2	2
Sherardia arvensis L.	Rubiaceae	1	1
Sinapis arvensis L.	Brassicaceae	4	6
Smyrnium perfoliatum L.	Apiaceae	2	6
Sonchus asper (L.) Hill	Asteraceae	4	10
Teucrium capitatum L.	Lamiaceae	1	1
Thymbra capitata (L.) Cav.	Lamiaceae	3	15
Thymbra spicata L.	Lamiaceae	1	1
Tordylium apulum L.	Apiaceae	2	2
Trifolium angustifolium L.	Fabaceae	1	1
Trifolium campestre Schreb.	Fabaceae	2	3
Trifolium lucanicum Guss.	Fabaceae	1	1
Urospermum picroides (L.) Scop. ex F.W.Schmidt	Asteraceae	1	1
Valerianella discoidea Loisel.	Caprifoliaceae	4	6
Vitex agnus-castus L.	Verbenacheae	2	14

Table S6: Nestedness contribution scores for each plant species visited by hoverflies in each visitation network. For details, see Materials and Methods. Empty cells denote absence of the species from the respective network.

	**2013**			**2014**			**2015**		
**Plant**	**Core**	**Perimeter**	**Unburnt**	**Core**	**Perimeter**	**Unburnt**	**Core**	**Perimeter**	**Unburnt**
**Amaryllidaceae**									
Allium neapolitanum	0.546								
Allium exile									-0.753
**Apiaceae**									
Bupleurum gracile	0.509	-0.360			0.739				
Daucus carota	-0.795	0.594	1.371	-0.964	1.717	-0.895	1.271	1.464	0.240
Eryngium campestre	0.081								
Pimpinella cretica	0.244		0.632	0.114					-0.100
Pimpinella peregrina			-0.380			0.438			-0.394
Scaligeria napiformis		-0.667			-0.072				
Scandix pecten-veneris									-0.687
Smyrnium perfoliatum						0.111			-0.753
Tordylium apulum	0.395						-1.044		
**Asparagaceae**									
Bellevalia trifoliata	0.241								
Leopoldia comosa						-0.509			
Muscari commutatum	-0.137								
Ornithogalum montanum							0.121		
**Asphodelaceae**									
Asphodelus ramosus		0.112							
**Asteraceae**									
Anthemis chia	-0.756		-0.736						
Anthemis cretica		0.432							
Centaurea urvillei	0.501								
Crepis commutata	1.462	1.399		1.954	1.441	0.817	1.295		
Crepis multiflora				0.207	-0.392				
Glebionis coronaria						-0.929			
Helichrysum stoechas	-0.353	-0.545	0.020	-0.754	-0.538		2.008	0.306	
Hypochaeris achyrophorus	-0.674			0.367	0.409				

	2013			2014			2015		
**Plant**	**Core**	**Perimeter**	**Unburnt**	**Core**	**Perimeter**	**Unburnt**	**Core**	**Perimeter**	**Unburnt**
Leontodon tuberosus	-0.353			0.531	0.347		0.618		
Pallenis spinosa							0.782		-0.774
Picris rhagadioloides		0.140	-0.185	-0.041	1.269				
Podospermum canum				0.619					
Scorzonera elata				0.269			0.733		
Sonchus asper				1.692	1.104			1.301	
Urospermum picroides				0.007					
**Boraginaceae**									
Anchusa undulata									-0.774
**Brassicaceae**									
Biscutella didyma				-0.522					
Hirschfeldia incana		2.337		0.769	0.567				
Sinapis arvensis					1.053				
**Caprifoliaceae**									
Valerianella discoidea	1.097								
**Cistaceae**									
Cistus creticus			-0.612	2.159	-0.171		0.233	1.035	
Cistus parviflorus					-0.981	-0.403		1.165	-0.801
Cistus salviifolius	-0.960				-1.035			1.034	
Fumana arabica				0.606		0.035			
Fumana thymifolia						1.216			-0.735
**Convolvulaceae**									
Convolvulus althaeoides				1.054	1.088				
**Euphorbiaceae**									
Euphorbia acanthothamnos				-1.106			-0.175		
**Fabaceae**									
Anthyllis hermanniae								0.406	
Genista acanthoclada			-0.593	-1.093					
Lotus ornithopodioides					0.963				
Ononis reclinata	0.577								
Trifolium campestre	-0.705								

	2013			2014			2015		
**Plant**	**Core**	**Perimeter**	**Unburnt**	**Core**	**Perimeter**	**Unburnt**	**Core**	**Perimeter**	**Unburnt**
Trifolium angustifolium									-0.690
Trifolium lucanicum				-1.007					
**Gentianaceae**									
Centaurium tenuiflorum					1.029				
**Geraniaceae**									
Geranium purpureum		0.131							
**Hypericaceae**									
Hypericum empetrifolium		0.042			1.054				
**Lamiaceae**									
Lamium amplexicaule		-1.163							
Origanum onites	0.235		0.625			1.500			-0.195
Salvia viridis	0.884								
Salvia fruticosa		-1.088				-1.258	-0.745		
Satureja thymbra			-0.835			-1.047			
Teucrium capitatum								0.427	
Thymbra capitata	-0.276		-0.871	-0.984		0.509	0.489		0.059
Thymbra spicata		0.325							
Vitex agnus-castus	-0.258						0.690		
**Liliaceae**									
Gagea graeca	0.244								
**Primulaceae**									
Anagallis arvensis	1.117	-1.125							
**Rubiaceae**									
Sherardia arvensis		0.185							
**Rutaceae**									
Ruta chalepensis							-0.759		

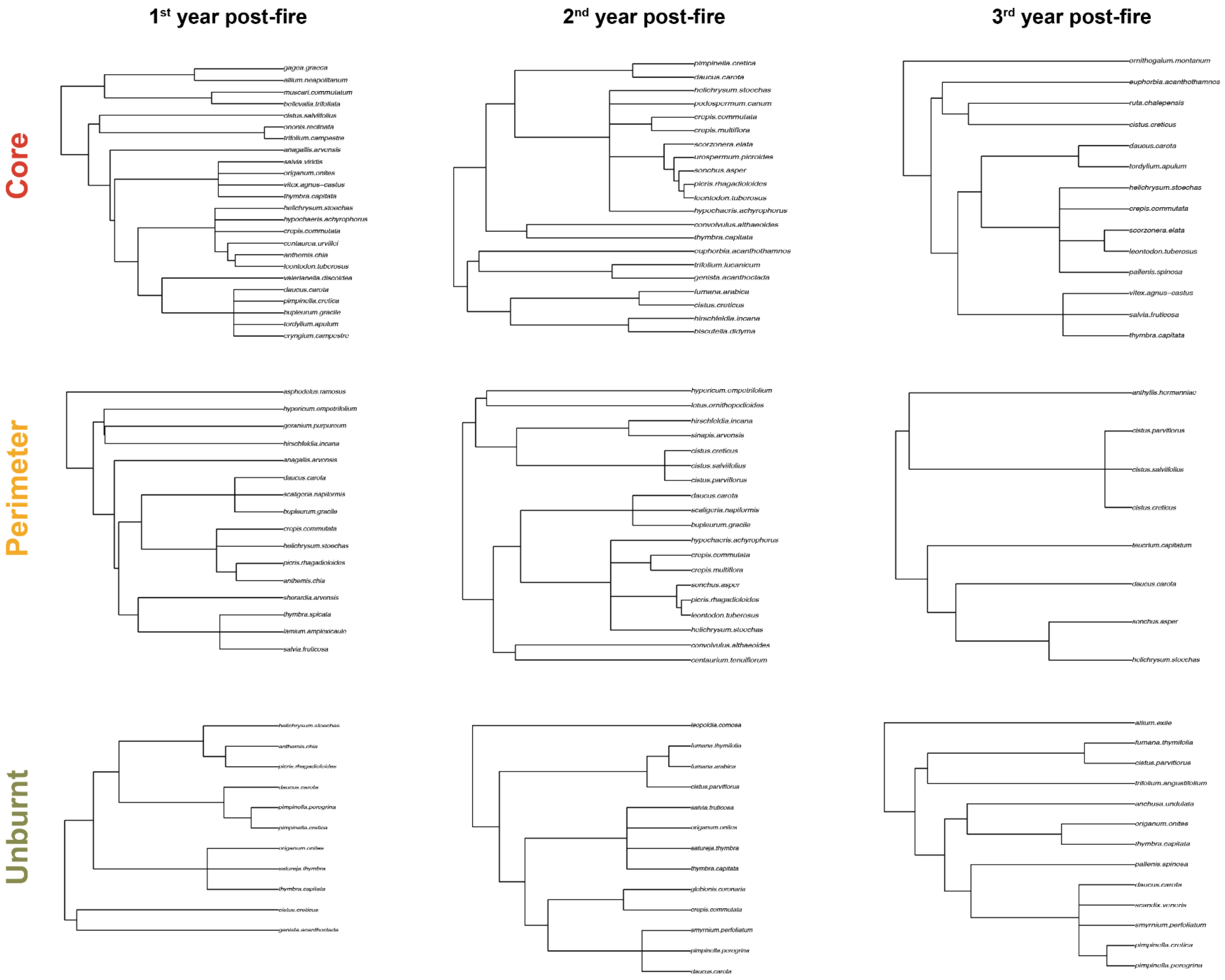

Figure S1: The phylogenetic trees of the hoverfly host plants for the three post fire years in the three site categories
